# Amelioration of Parkinsonian tremor evoked by DBS: which role play cerebello-(sub)thalamic fiber tracts?

**DOI:** 10.1007/s00415-023-12095-1

**Published:** 2023-11-30

**Authors:** Daniel Deuter, Tobias Mederer, Zacharias Kohl, Patricia Forras, Katharina Rosengarth, Mona Schlabeck, Daniela Röhrl, Christina Wendl, Claudia Fellner, Nils-Ole Schmidt, Jürgen Schlaier

**Affiliations:** 1 Department of Neurosurgery, University Hospital Regensburg, Franz-Josef-Strauß-Allee 11, 93053 Regensburg, Germany; 2 Center for Deep Brain Stimulation, University Hospital Regensburg, Franz-Josef-Strauß-Allee 11, 93053 Regensburg, Germany; 3 Department of Neurology, University Hospital Regensburg, Franz-Josef-Strauß-Allee 11, 93053 Regensburg, Germany; 4 Department of Neurology, Regensburg Medbo District Hospital, Universitätsstraße 84, 93053 Regensburg, Germany; 5 Department of Anesthesiology, University Hospital Regensburg, Franz-Josef-Strauß-Allee 11, 93053 Regensburg, Germany; 6 Department of Radiology, University Hospital Regensburg, Franz-Josef-Strauß-Allee 11, 93053 Regensburg, Germany; 7 Department of Radiology, Regensburg Medbo District Hospital, Universitätsstraße 84, 93053 Regensburg, Germany

**Keywords:** DBS, Parkinson’s disease, Movement disorders, Functional network, Probabilistic tractography, Dentato-rubro-thalamic tract

## Abstract

**Background:**

Current pathophysiological models of Parkinson’s disease (PD) assume a malfunctioning network being adjusted by the DBS signal. As various authors showed a main involvement of the cerebellum within this network, cerebello-cerebral fiber tracts are gaining special interest regarding the mediation of DBS effects.

**Objectives:**

The crossing and non-decussating fibers of the dentato-rubro-thalamic tract (c-DRTT/nd-DRTT) and the subthalamo-ponto-cerebellar tract (SPCT) are thought to build up an integrated network enabling a bidimensional communication between the cerebellum and the basal ganglia. The aim of this study was to investigate the influence of these tracts on clinical control of Parkinsonian tremor evoked by DBS.

**Methods:**

We analyzed 120 electrode contacts from a cohort of 14 patients with tremor-dominant or equivalence-type PD having received bilateral STN-DBS. Probabilistic tractography was performed to depict the c-DRTT, nd-DRTT, and SPCT. Distance maps were calculated for the tracts and correlated to clinical tremor control for each electrode pole.

**Results:**

A significant difference between “effective” and “less-effective” contacts was only found for the c-DRTT (*p* = 0.039), but not for the SPCT, nor the nd-DRTT. In logistic and linear regressions, significant results were also found for the c-DRTT only (*p*_model logistic_ = 0.035, *p*_tract logistic_ = 0,044; *p*_linear_ = 0.027).

**Conclusions:**

We found a significant correlation between the distance of the DBS electrode pole to the c-DRTT and the clinical efficacy regarding tremor reduction. The c-DRTT might therefore play a major role in the mechanisms of alleviation of Parkinsonian tremor and could eventually serve as a possible DBS target for tremor-dominant PD in future.

## Introduction

Current pathophysiological models of movement disorders suggest malfunctioning networks consisting of specific cortical and subcortical areas rather than isolated local defects in the brain [1–5]. Similarly, the mechanistic understanding of how deep brain stimulation (DBS) works in these conditions, evolved away from rather local lesional thinking toward network-based theories. As DBS effects have not only been found at the location of the electrode pole itself, but also in connected regions [6–9], it is thought that the DBS signal interacts with these malfunctioning networks and transfers them into a better working state [1, 10].

Subcortical fibers are thought to be electrically influenced by the DBS signal inside a volume of tissue electrically activated (VTA) [11, 12]. As these fiber tracts are probably mediating DBS effects inside the brain, special interest has been paid to subcortical fiber tracts with regard to DBS [13–20].

In Parkinson’s disease (PD), a malfunctioning functional network mainly consisting of the basal ganglia, the thalamus, cerebral cortex, and the cerebellum is assumed to play a role in the manifestation of the cardinal symptoms tremor, rigidity, and bradykinesia. More specific, this network is thought to include the areas of the external and internal globus pallidus (GPe/GPi), striatum, primary motor cortex (M1), (pre-) supplementary motor area (preSMA/SMA), pedunculopontine nucleus (PPN), pontine nuclei, the subthalamic nucleus (STN), and the substantia nigra [2]. Various authors recently underlined especially an important role of the cerebellum inside this functional network [21–23]. Cerebellar–cerebral fiber tracts are therefore gaining special interest with regard to a possible mediation within this network [15, 24–27].

The dentato-rubro-thalamic tract (DRTT), connecting the cerebellum with the thalamus, consists of crossing and non-decussating fibers (c-DRTT and nd-DRTT) and has been shown to mediate tremor-reducing DBS effects in essential tremor (ET) [18–20, 28]. Some authors even proposed the DRTT, representing the main cerebellar efferent pathway [29, 30], as a common structure mediating tremor-reducing DBS effects irrespective of the underlying pathology. Because many of the currently used DBS targets for tremor are located along the DRTT, stimulation of the tract rather than the targets themselves was discussed [15–17, 31].

**Fig. 1:**
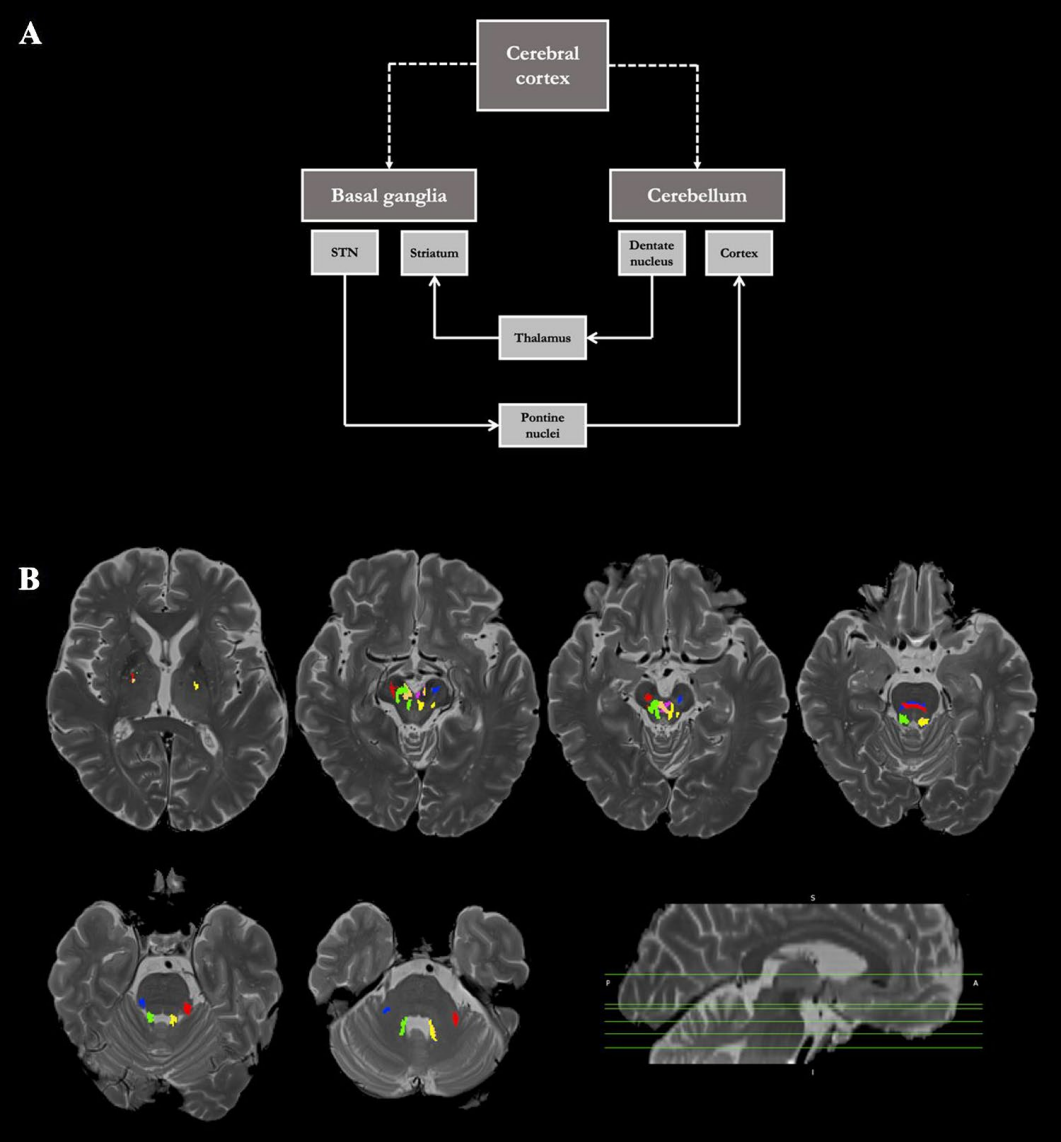
**A** Model of a two-dimensional integrated network between the cerebellum and the basal ganglia via a subthalamo-cerebellar and a dentato-thalamo-striatal fiber tract as initially proposed by Bostan et al. [32]. The SPCT represents the main cerebellar afference, whereas the DRTT, consisting of a crossing and a non-decussating part, is regarded as the main efference of the cerebellum. Figure adopted from Bostan et al. [32]. **B** Course of the nd-DRTT (green/yellow), c-DRTT (orange/pink), and SPCT (red/blue). Axial slices are shown on the level of the foramen of Monroi, cerebral peduncle, crossing of the c-DRTT, crossing of the SPCT, superior cerebellar peduncle (SCP), and middle cerebellar peduncle (MCP). The sagittal image below right shows the position of the axial slices. The DRTT ascends from the dentate nucleus through the SCP to the thalamic nuclei, partially crossing in the mesencephalic area [29, 33]. The SPCT descends from the STN, decussating at the level of the pons [24, 26, 32], and enters the cerebellum through the MCP

A second main cerebello-cerebral fiber tract is the subthalamo-ponto-cerebellar tract (SPCT), descending from the STN to the cerebellum [24, 26, 32]. The SPCT represents one of the main afferent fiber tracts carrying information from the basal ganglia to the cerebellum [26]. Bostan et al. [32] first described this tract in cebus monkeys and presented a model of a system enabling a bidimensional cerebello-basal ganglia interaction within an integrated network of this subthalamo-cerebellar tract and a dentato-thalamo-striatal fiber tract, as depicted in Fig. 1A. For the detailed course of the DRTT and the SPCT, representing the main white matter components within this integrated network, please see Fig. 1B.

The aim of this study was to investigate the influence of these cerebello-cerebral fiber tracts on clinical control of Parkinsonian tremor induced by DBS.

## Methods

We used a workflow for probabilistic tractography to depict the c-DRTT, the nd-DRTT, and the SPCT in patients with Parkinsonian tremor and correlated results from neurological single pole testing with the distances to these tracts. A total of 120 electrode poles in 14 patients with tremor-dominant or equivalence-type PD was analyzed. The study was approved by the local ethics committee of the University Hospital of Regensburg (protocol code Z-2017-0876-10) and was performed in accordance with the Declaration of Helsinki.

### Cohort

Patients suffering from tremor-dominant or equivalence-type PD having received bilateral STN-DBS at our center between 2015 and 2020 were screened for inclusion. Patients with insufficient pre- or postoperative imaging were excluded (*n* = 1) as well as patients with missing or insufficient clinical protocols (*n* = 8). The final cohort consisted of 14 patients with 28 leads implanted leading to a total of 120 electrode contacts. Clinical and demographical information as well as information on implanted leads and impulse generators (IPGs) are shown in Table 1.

**Table 1:** Clinical and demographic information of the patients’ cohort including information on implanted leads and IPGs

Sex	Male	*N* = 11
	Female	*N* = 3
Age	Mean age at the time of surgery	63.29 years (95% CI 61,628–64,943)
Duration of illness	Mean duration of illness at the time of surgery	8.64 years (95% CI 7223–10,062)
Implanted leads	Abbott 6170 (Abbott Laboratories, North Chicago, IL)	*N* = 8
	Medtronic 3389 (Medtronic plc, Dublin, Ireland)	*N* = 5
	Boston Scientific DB-2201-30AC (Boston Scientific, Marlborough, MA)	*N* = 1
Implanted IPG	Abbott infinity	*N* = 8
	Medtronic Activa RC	*N* = 3
	Medtronic Activa PC	*N* = 2
	Boston Scientific Vercise	*N* = 1
l-DOPA equivalence dose	Mean dose preoperative	757.14 mg (95% CI 675,083–839,202)
UPDRS score in l-DOPA off	Mean UPDRS score	39.04 (95% CI 36,274–41,803)
UPDRS score in l-DOPA on	Mean UPDRS score	18.73 (95% CI 16,556–20,906)

### Imaging

To avoid movement artifacts, preoperative imaging was performed with patients under general anesthesia at a 3 T Magnetom Skyra scanner (Siemens Healthcare, Erlangen, Germany). Imaging parameters were used as previously described by Strotzer et al. [34]. MR imaging included a T1-weighted sequence + one and a half times dose of Gadolinium to visualize crucial blood vessels, a T2-weighted sequence parallel to the AC-PC line to visualize relevant subthalamic structures for targeting and a DTI sequence with 64 gradient directions. DTI imaging included b0 images as well as a b0 image with inverted phase-encoding direction (posterior to anterior, “P2A”). Targets were defined in the dorsolateral aspect of the STN based on the Beijani line [35] at the anterior border of the red nucleus at its biggest expansion. At the day of surgery, a CT scan with a CRW head ring (Integra Radionics, Burlington, VT) mounted on the patient’s head was performed for definition of stereotactic trajectories at a Somatom Definition Flash scanner (Siemens Healthcare, Erlangen, Germany). MR and CT imaging was fused with Brainlab^®^ iPlanNet 3.0 (BRAINLAB, Munich, Germany). Trajectories were defined avoiding blood vessels, sulci, and eloquent neuronal structures.

### Surgical procedure

Surgery was performed with patients awake. Long-acting antiparkinsonian drugs like dopamine agonists, catechol *O*-methyltransferase (COMT) inhibitors, or monoamine oxidase (MAO) inhibitors were stopped 3 to 4 days prior to surgery and action was bridged using l-Dopa. Microelectrode recording was performed with up to five trajectories. At areas with good electrophysiological STN signal, stimulation was performed with non-permanent macroelectrodes for intraoperative testing. The position of the final electrode was chosen based on intraoperative clinical testings at positions with wide therapeutic windows. Positions of permanent electrodes were verified with intraoperative X-rays in overlay with X-rays of the non-permanent testing electrode. IPGs and lead extensions were implanted in a second surgery with patients under general anesthesia.

### Postoperative workup

Postoperatively, CT scans with 1 mm slice thickness were performed to verify the positions of the electrodes and to exclude relevant bleeding. Antiparkinsonian medication was postoperatively started again as preoperatively taken. Neurological monopolar single pole testing was performed at least 30 days after surgery to avoid transient microlesional effects. A fixed frequency of 130 Hz and a fixed pulse width of 60 µs were used for testings. Tremor was scaled on the Fahn–Tolosa–Marin tremor rating scale [36] from 0 to 4. Tremor reduction was rated on a five-parted scale (0%, 25%, 50%, 75%, and 100%) by neurologists specialized in movement disorders. Final stimulation parameters were chosen based on these clinical testings. In the further course, antiparkinsonian medication dosages and DBS stimulation settings were titrated depending on clinical response. Within this study, we analyzed results regarding rest tremor from the neurological single pole testing 30 days postoperatively.

### Probabilistic tractography

As deterministic tractography, which calculates only one main vector for each imaging voxel and has therefore limitations in visualizing crossing, kissing, and fanning fibers, probabilistic tractography was used. Using probabilistic tractography, fiber tracking is rather based on probability distributions within each voxel, and is therefore regarded as the gold standard in tractographic approaches [37–40].

We performed probabilistic tractography using FSL 6.0.3 [41–43] including protocols for brain extraction [44], correction of eddy-current induced errors as well as proband movement errors [45] and susceptibility-induced errors [43, 46]. Models of diffusion orientations were calculated for each imaging voxel with BedpostX [47] based on a workflow proposed by Behrens et al. [37] using Markov Chain Monte Carlo sampling. Subsequently, streamlines between seed and targets regions were calculated based on these data using ProbtrackX2 (cval = 0.2, Sval = 2000, stepl = 0.5, Pval = 5000, fibt = 0.01, distt = 0.0, sampvox = 0.0) evoking track probability maps for each of the analyzed fiber tracts [47]. To depict the specific fiber tracts, ROIs were defined on the T2 images, which were co-registered to the DTI sequences. For tractography of the c-DRTT and the nd-DRTT, the superior cerebellar peduncle (SCP) was defined as a seed region with a target in the ipsi- or contralateral Ncl. ruber and the lateral interspace to the STN. For delineation of the SPCT, ROIs were adopted from Lipp et al. [26]: As a seed region, we used a supramarginal segmentation of the STN with some surrounding tissue marked on a coronal plane located approximately at the level of the Beijani line or ventral of it as a seed region. The contralateral middle cerebellar peduncle (MCP) served as a target region; the ipsi- and contralateral SCP as exclusion regions.

Subsequently, tracts were binarized based on the probability maps. Several methods how to choose the threshold for the final definition of fiber tracts exist as previously described [20]. As thresholds based on 200%, 400%, 600%, 800%, and 1000% of the robust range ignoring extreme spikes within the histogram (“-thrp”) showed best results in previous tests, tracts were calculated based on these values and were compared in direct overly on the T2-weighted images in parallel. For the DRTT, the threshold providing the best discrimination between the two parts of the DRTT was chosen. As a single value was chosen for each subject in general instead of for each hemisphere, the threshold partially represents compromises between the two hemispheres. For the SPCT, a second threshold was chosen. Automatized distance maps, providing maps with the distance to each of the specific tracts as a value within each voxel were calculated using ANTs 2.2.0 [48].

### Lead localization and export of distances of electrode poles to the fiber tracts

Leads were reconstructed using LeadDBS [49] and visualized in native patient space (co-registration CT to MRI ANTs [50], MRI to MRI SPM [51] or FSL FLIRT [52, 53], normalization three-step affine normalization ANTs [50, 54, 55], pre-reconstruction PaCER [56], and manual localization).

**Fig. 2:**
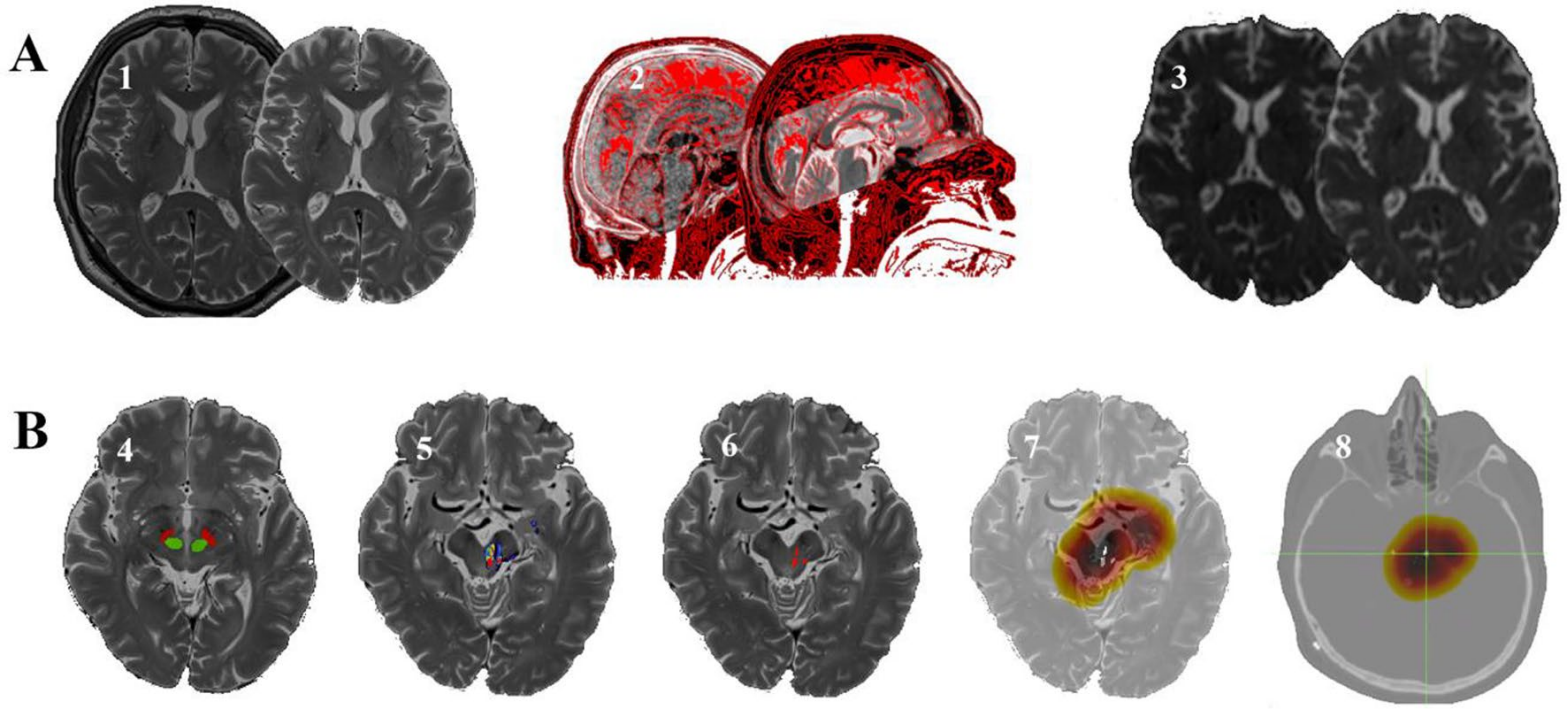
**A** Different steps of preprocessing: From left to right: (1) brain extraction, (2) co-registration of CT and MRI, (3) correction of eddy-current induced errors, proband movement errors, and susceptibility-induced errors. **B** Different steps of processing: (4) definition of seed and target regions, (5) calculation of probability maps, (6) tract binarization, (7) calculation of distance maps, and (8) export of distances based on the distance maps for the pole coordinates of the DBS electrode

T2-weighted images were co-registered to the postoperative CT scan. A transformation matrix was calculated for this fusion and was applied to all tractographic results using FSL FLIRT [52, 53] to co-register data from FSL to LeadDBS. Coordinates of electrode poles were exported using Matlab 2018a (MathWorks, Natick, MA) and visually controlled to lie on the electrode in the postoperative CT scan. For these coordinates, distances to the three tracts of interest were exported based on calculated distance maps. If distances of the pole to the tracts were larger than 10 mm, we adopted thresholds one step lower to gain more realistic representations of fiber tracts. Steps of preprocessing and processing are summarized in Fig. 2A and B.

### Statistics

We defined groups of “effective” and “less-effective” contacts based on a coefficient Coeff_Tremorred_ defined as the current or voltage [mA or V] divided through the derived tremor reduction [%] regarding resting tremor. To additionally depict the absolute amount of tremor reduction rather than the relative amount, a further coefficient Coeff_TremorredAbs_ was defined as the current or voltage divided through the product of the relative tremor reduction [%] and the absolute amount of baseline resting tremor on the Fahn–Tolosa–Marin scale. Mean values were calculated for each electrode pole to exclude bigger influences of poles with more testing steps due to a wide therapeutic window. In segmented poles (32 of the electrode contacts were segmented poles divided into three segments each leading to a total of 96 segments), only ring stimulation was analyzed due to inadequate comparability between stimulation parameters of segmented leads and ring poles. As stimulation of poles with 1 mA leads to comparable VTAs as stimulation with 1 V assuming fixed impedances [57], current- and voltage-steered IPGs were analyzed together. Groups of “effective” and “less-effective” contacts were dichotomized based on the median values of Coeff_Tremorred_ or Coeff_TremorredAbs_ (‘median split’) leading to equally sized groups.

Differences in distances to the nd-DRTT, the c-DRTT and SPCT between the groups were analyzed using independent sample t tests. Additionally, logistic regression analyses were performed as well as linear regressions assuming a linear correlation. *p* values < 0.05 were regarded statistically significant. Statistics were calculated using SPSS 25 (IBM, Armonk, NY).

## Results

### *T* tests

Regarding the absolute amount of tremor reduction (Coeff_TremorredAbs_), a significant difference of mean distances between effective and less-effective contacts was found only for the c-DRTT (mean value ± standard deviation 3.78 ± 1.49 mm vs. 4.61 ± 1.92 mm; *p* = 0.039). Effective contacts were located closer to the c-DRTT than less-effective contacts. Although, in mean, contacts were closest to the SPCT and farthest away from the c-DRTT, no significant differences were found for the SPCT nor the nd-DRTT. Results are depicted in Table 2A. Between “effective” and “less-effective” contacts, classified based on Coeff_Tremorred_, no significant differences were found.

**Table 2:** Results from T tests (A), logistic regression (B) and linear regression analyses (C)

(A) Tract	“Effective” contacts	“Less-effective” contacts	*p* value (two-sided)
nd-DRTT	3.48 ± 1.23 mm	3.29 ± 1.22 mm	0.484
c-DRTT	3.78 ± 1.49 mm	4.61 ± 1.92 mm	0.039
SPCT	2.55 ± 0.99 mm	2.53 ± 1.12 mm	0.930

(B) Tract	Exp(*B*)	Nagelkerke *R*^2^	*p* value model	*p* value tract
nd-DRTT	0.874	0.009	*p*_model_ = 0.476	*p*_nd-DRTT_ = 0.478
c-DRTT	1.336	0.075	*p*_model_ = 0.035	*p*_c-DRTT_ = 0.044
SPCT	0.981	< 0.001	*p*_model_ = 0.928	*p*_SPCT_ = 0.928

(C) Tract	Constant	Unstandardized B	*R*^2^	*p* value tract and model
nd-DRTT	3.579	− 0.170	0.008	*p*_ndDRTT_ = 0.450
c-DRTT	1.568	0.342	0.064	*p*_cDRTT_ = 0.027
SPCT	2.669	0.132	0.003	*p*_SPCT_ = 0.615

(A) Mean distances to the specified tracts ± standard deviations. Additionally, results from independent samples *t* tests with two-sided *p* values are shown. (B) Results from logistic regression analyses for the specified tracts. Exp(*B*) and Nagelkerke *R*^2^ are shown as well as the *p* value for the statistic model and for the specific tract. (C) Results from linear regression analyses for the specific tracts assuming a linear correlation between the distances to the specific tracts and Coeff_TremorredAbs_. Exp(*B*) and *R*^2^ are shown as well as *p* values

### Logistic regression

**Fig. 3:**
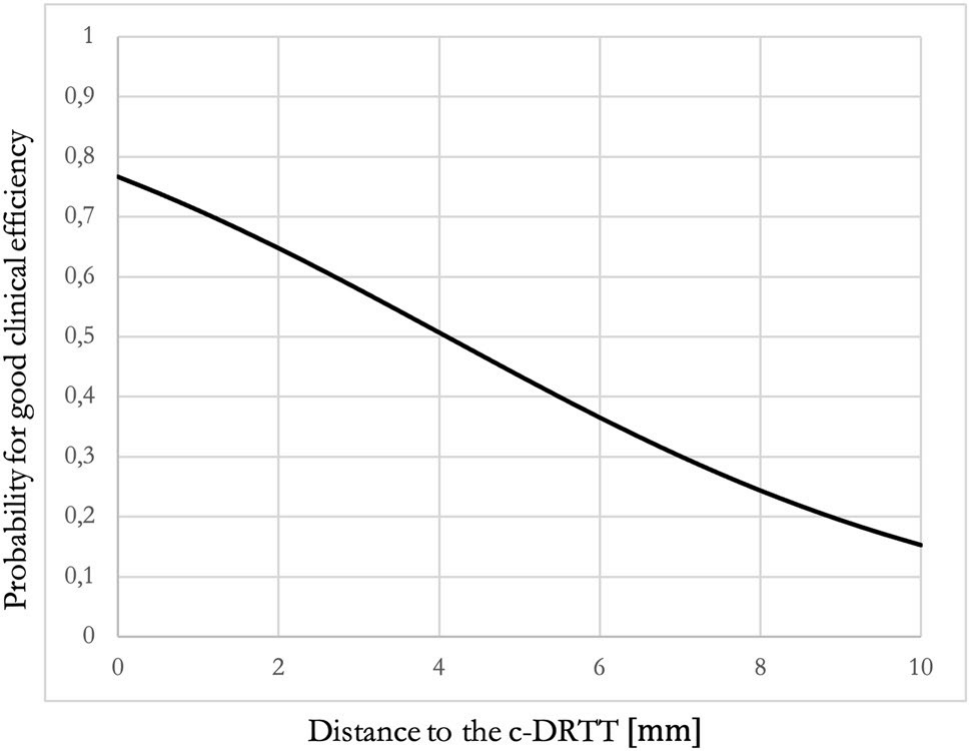
Results from logistic regression for the c-DRTT (predictive model): the graph shows the term 1 − (*e*^logit^/1 + *e*^logit^) with logit = constant + *β*0 * *x* representing a predictive model for the probability for good clinical efficacy of an electrode contact in relation to the distance to the c-DRTT. The c-DRTT was the only tract, for which a significant influence was found in logistic regression analyses

In logistic regression analyses of groups binarized based on Coeff_TremorredAbs_, significant results were found only for the c-DRTT (Exp(B) = 1.336; Nagelkerke *R*^2^ = 0.075; *p*_model_ = 0.035; *p*_c-DRTT_ = 0.044). Results of the logistic regression and the predictive model are depicted in Table 2B and Fig. 3. Logistic regression analyses of groups based on Coeff_Tremorred_ were not statistically significant.

### Linear regression

Assuming a linear correlation between the distances to the specific tracts and Coeff_TremorredAbs_, a significant correlation was only found for the c-DRTT in linear regressions, as shown in Table 2C (unstandardized *B* = 0.342; *R*^2^ = 0.064; *p* = 0.027). Linear regression analyses between distances to the specific tracts and Coeff_Tremorred_ showed no statistically significant results.

## Discussion

**Fig. 4:**
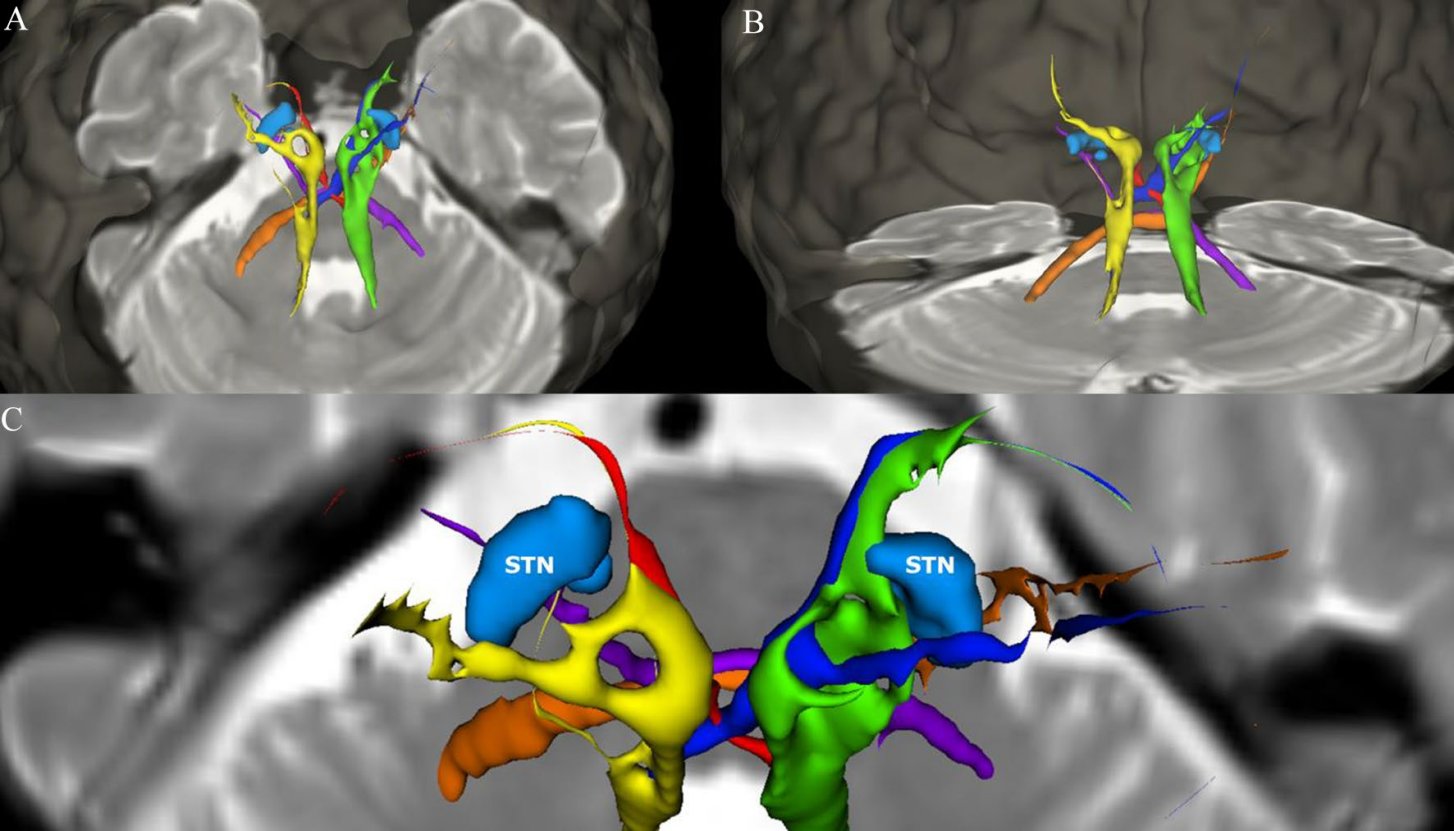
Anatomical course of the tracts of interest within this study and their spatial position relatively to the STN in the three-dimensional space: Results from probabilistic tractography including the nd-DRTT (yellow/green), the c-DRTT (red/dark blue), and the SPCT (orange/violet) are shown for an exemplarily patient in relation to an axial T2-image on the level of the MCP and the semi-transparent cortical surface as well as the STN (light blue). **A** View from posterior-superior, **B** view from posterior, and **C** view from superior showing the position of the STN relatively to the surrounding fiber tracts. Please be aware that measurements used for statistical analyses were not performed on post-processed and smoothed data meant for visualization only, but on raw data

We evaluated a total of 120 electrode poles in 14 patients suffering from tremor-dominant or equivalence-type PD. A significant correlation between the distances to these specific tracts and clinical efficacy was only found for the c-DRTT, but not for the nd-DRTT and the SPCT. Figure 4 shows the anatomical course of these tracts as well as their spatial position relatively to the STN.

All of these fiber tracts have previously been discussed with respect to the mediation of tremor-reducing DBS effects in PD [14, 15, 24, 26, 27]. Abdulbaki et al. [27] found effective contacts being significantly closer to the DRTT than non-effective contacts in 60 hemispheres of patients with Parkinsonian tremor, but the authors did not differentiate between the c-DRTT and the nd-DRTT. Another study also found better tremor control if electrode contacts were closer to the DRTT in a cohort of 36 patients with ET, PD, multiple sclerosis, dystonic head tremor, and tardive dystonia. The authors used the DRTT, depicted by deterministic tractography, as a DBS target and implanted leads into the thalamic or subthalamic area [15]. Additionally, two studies investigated both the DRTT and the SPCT. Lipp et al. [26] analyzed the anatomical course of the DRTT and the SPCT as well as microstructural properties of these tracts in PD patients compared to healthy controls. Additionally, Sweet et al. [24] found a non-significant trend for electrode poles located closer to the DRTT with good clinical tremor control, but reported no results for the SPCT. Also, in ET, the previous studies found a correlation between the distance of electrode contacts to the DRTT [18, 19, 28], especially to the c-DRTT [20].

Current pathophysiological models propose a large-scale functional network, including the basal ganglia, thalamus, the cerebral cortex, and the cerebellum [2]. Apart from the STN, which was used as the target for implantation of DBS leads in this study, different targets like the pedunculopontine nucleus, thalamic targets like the VIM, and the caudal zona inserta have been used [15, 31, 58]. This could underline the theory of a large-scale functional network spread all over the brain with different entry points for mediation of this network by the DBS signal. Cerebellar–cerebral fibers as a specific part of this functional network, forming a cortico-cerebellar and a cortico-basal ganglia network, have been discussed with respect to compensatory and causative alterations in PD patients [59]. Additionally, Muthuraman et al. found specific differences in the cerebello-cortical network when comparing patients with PD and ET as well as mimicked tremor [60]. As many of the targets currently used for DBS in tremor of different causes like the ventral intermediate nucleus of the thalamus (VIM), the STN, or the caudal zona inserta are located along the course of the DRTT, it has been discussed if the DRTT itself could be the structure actually tackled rather than the gray matter targets themselves [15–17, 31].

Apart from the small number of patients included, one of the limitations of this study is the necessary binarization of clinically “effective” and “less-effective” contacts. We used two coefficients Coeff_Tremorred_ and Coeff_TremorredAbs_, which depict the two aspects of clinical efficacy (the amount of tremor reduction and the current or voltage needed to reach this effect). Depending on the definition, many “semi-effective” contacts are getting grouped into the group of “effective” or “less-effective” contacts, leading to different results depending on the chosen method due to dichotomization based on the median value. Furthermore, in this study, we chose to calculate mean values for each electrode pole to not overestimate poles with more testing points due to wide therapeutic windows. Another possibility would be to analyze all testing points from the neurological single pole testing. Using this approach, if stimulation of a specific electrode pole with 0.5 mA led to a tremor reduction of 25%, 1 mA to 75% and 1.5 mA to 100%, all these points would have been analyzed as three independent testing points. Additionally, we only included data from ring-like stimulation, but not from segmented poles due to inadequate comparability of currents/voltages and better effects due to usually higher efficacy of these segmented poles. All electrode poles including current-steered and voltage-steered systems were investigated within one analysis as stimulation with 1V would lead to comparable VTAs as with 1mA according to current basic models assuming fixed impedances [57]. Another limitation is the lack of standardization for definition of thresholds, which provide the base for the binarization of the specific fiber tracts. We previously discussed this issue how to choose these thresholds as well as potential principles on which criteria this decision could be based [20]. Due to the analysis of only results from neurological single pole testing 30 days postoperatively, no final conclusions can be drawn regarding possible delayed tremor-reducing DBS effects due to the lack of a long-term follow-up.

The SPCT has been previously described in only a couple of studies. Bostan et al. [32] first presented a model of two fiber tracts enabling a bidimensional interaction between the basal ganglia and the cerebellum. Based on these data from cebus monkeys, another group of authors confirmed the presence of a dentato-thalamo-striato-pallidal and subthalamo-cerebellar tract in humans by probabilistic tractography [25]. Nevertheless, the description of the course of the SPCT varies in the literature as in two subsequent tractographic studies in humans, and the SPCT was described heading toward the SCP by one group [24], but to the MCP by the other group [26]. As Pelzer et al. [25] also found the subthalamo-cerebellar tract running through the MCP, we chose the MCP as a target including exclusion regions in the SCP to avoid depicting fibers of the DRTT for fiber tracking. Seed and targets region were adopted based on the publication by Lipp et al. [26] leading to reliably appearing fiber tracts, running via the cerebral peduncle through the STN and the subthalamic area with decussation in the pons and finally entering the cerebellum through the MCP.

In contrast to various previous authors, which used deterministic tractography [15, 24, 26], in this study, probabilistic tractography was used, which is able to depict crossing, fanning, and kissing fibers unlike deterministic approaches. Additionally, MR imaging from a 3T scanner with 64 gradient directions and patients under general anesthesia was used, providing excellent imaging quality. Due to the importance of small distances in the range of only several millimeters in DBS surgery, we additionally avoided normalization into a standard space due to possible normalization errors. Distances were measured and analyzed automatically in individual patients. Because of the spatially complex courses of the specific tracts and possible errors if measuring is performed for example only on axial slices or on manually located nearest points on the tract, the importance of automized measurements has to be clearly underlined. Still, all authors of preceding studies investigating cerebello-thalamic fiber tracts with respect to reduction of Parkinsonian tremor used manual measurements [15, 24, 27].

Mechanistically, different possibilities of how the DBS signal might influence the large-scale functional network via the DRTT could be discussed. Possible mechanisms include mediation of the “direct pathway” via the dorsal striatum, the GPi, and the thalamus; mediation of the “indirect pathway” via dorsal striatum, GPe, STN, GPi, and the thalamus [61], and a modulation of cerebellar in- and output signals. It has to be kept in mind, that within this study, only Parkinsonian tremor was analyzed as one of the three cardinal symptoms of PD, but not bradykinesia or rigidity. Therefore, the dorsolateral part of the STN, currently probably the most widely used DBS target in PD, might still represent the best spot for patients with multi-dimensional PD symptoms as also previously stated by Hariz et al. [62]. Additionally, the limbic, associative and cognitive projections of the medial STN [62, 63] and other neuroanatomic structures in spatial proximity to the DRTT have to be taken into account, which might mediate side-effects of DBS that were not part of this study.

In summary, we found a significant correlation between the distance of the DBS electrode pole to the c-DRTT and the clinical efficacy of these poles regarding tremor reduction. Even if contacts were in mean closer to the SPCT, for this tract, no significant influence was found, nor for the nd-DRTT. These data might help to better characterize the functional network underlying the PD disease, even if this study only focused on Parkinsonian tremor as only one of the three cardinal symptoms of PD. For the alleviation of the other main symptoms like rigidity and bradykinesia, different structures could play a role instead. Nonetheless, for the final definition of the DRTT as a new DBS target for treatment of tremor-dominant PD, further prospective studies directly comparing DRTT-DBS and DBS of other targets like the STN or thalamic targets are needed.

## Data Availability

Data supporting the findings of this study are available from the corresponding author upon reasonable request.
